# Sequential Turnovers of Sex Chromosomes in African Clawed Frogs (*Xenopus*) Suggest Some Genomic Regions Are Good at Sex Determination

**DOI:** 10.1534/g3.116.033423

**Published:** 2016-09-07

**Authors:** Benjamin L. S. Furman, Ben J. Evans

**Affiliations:** Department of Biology, McMaster University, Hamilton, Ontario L8S 4K1, Canada

**Keywords:** sex chromosomes, *Xenopus*, rapid evolution, sex chromosome turnover, *SOX3*, Genetics of sex

## Abstract

Sexual differentiation is fundamentally important for reproduction, yet the genetic triggers of this developmental process can vary, even between closely related species. Recent studies have uncovered, for example, variation in the genetic triggers for sexual differentiation within and between species of African clawed frogs (genus *Xenopus*). Here, we extend these discoveries by demonstrating that yet another sex determination system exists in *Xenopus*, specifically in the species *Xenopus borealis*. This system evolved recently in an ancestor of *X. borealis* that had the same sex determination system as *X. laevis*, a system which itself is newly evolved. Strikingly, the genomic region carrying the sex determination factor in *X. borealis* is homologous to that of therian mammals, including humans. Our results offer insights into how the genetic underpinnings of conserved phenotypes evolve, and suggest an important role for cooption of genetic building blocks with conserved developmental roles.

For nearly all vertebrates, two sexes are needed to secure the benefits of genetic recombination associated with sexual reproduction (Barton and Charlesworth 1998). It is, therefore, not surprising that the genetic control of sexual differentiation is tightly regulated, and has remained unchanged for millions of years in several lineages (Graves and Peichel 2010; O’Meally *et al.* 2012; Veyrunes *et al.* 2008; Matsubara *et al.* 2006). However, genetic control of sexual differentiation has diversified in some groups. For example, nonhomologous sex chromosomes have been detected in several closely related species or populations of stickleback (Ross *et al.* 2009), medaka (Myosho *et al.* 2015), and cichlid (Roberts *et al.* 2009) fish, and rampant turnover of the sex chromosomes occurred over a broader phylogenetic scope in fish (Devlin and Nagahama 2002; Mank *et al.* 2006), gecko lizards (Gamble *et al.* 2015), and amphibians (Evans *et al.* 2012).

Among these turnover events, common elements have been independently coopted for sex determination in several instances. For example, one syntenic block of genes independently became sex-linked in a lizard (*Gekko hokouensis*) and birds (Kawai *et al.* 2009), and another separately became sex-linked in a frog (*Rana rugosa*) and therian mammals (Wallis *et al.* 2007; Uno *et al.* 2008, 2013). In addition, individual genes with sex-related function have repeatedly evolved into the trigger for sexual differentiation. Examples include homologs of *doublesex and mab-3 related transcription factor 1* (*DMRT-1*), an important sex-related gene in vertebrates (Zarkower 2001), which are triggers for sex determination in medaka fish, *Oryzias latipes* (Kondo *et al.* 2003, 2004), the African clawed frog *Xenopus laevis* (Yoshimoto *et al.* 2008), probably the Chinese half-smooth tongue sole (Chen *et al.* 2014), and all birds (Smith *et al.* 2009; but see Zhao *et al.* 2010). Similarly, homologs of *SOX3*, which is another important sex-related gene (Weiss *et al.* 2003), independently became triggers for sexual differentiation in the fish *O. dancena* (Takehana *et al.* 2014) and in the ancestor of therian mammals (Koopman *et al.* 1991). Turnover of sex chromosomes and the genes involved with sex determination provide opportunities to study how tightly regulated systems evolve, and in particular the extent to which this involves convergence, reversion to an ancestral state, or origin of genetic novelty.

In addition to being model organisms for biology (Cannatella and de Sá 1993; Hellsten *et al.* 2010; Harland and Grainger 2011), African clawed frogs (genus *Xenopus*) offer a promising system with which to study sex chromosomes. At least two species, *X*. *laevis* (Daudin 1802) and *X*. (*Silurana*) *tropicalis* (Gray 1864), have a nonhomologous trigger for sex determination (Yoshimoto *et al.* 2008; Olmstead *et al.* 2010; Roco *et al.* 2015). These two species are members of different subgenera that are distinguished from each other by the number of chromosomes (*x*) carried by the gametes of their respective diploid ancestors, *i.e.*, *x* = 10 for subgenus *Silurana* and *x* = 9 for subgenus *Xenopus* (Evans *et al.* 2015). All extant species in subgenus *Xenopus* are polyploid, but with disomic chromosomal inheritance, and tetraploids in this subgenus have 4*x* = 36 chromosomes. In *X. laevis*, a gene called *DM-W* is the master sex regulator of sex determination (Yoshimoto *et al.* 2008); this gene appeared in an ancestor of *X. laevis* after divergence from the ancestor of *X. tropicalis*, and is present in many close relatives of *X. laevis* (Bewick *et al.* 2011). In subgenus *Silurana*, *X. tropicalis* has a complex trigger for sex determination that resides on Y, W, and Z chromosomes (Roco *et al.* 2015). This system in *X. tropicalis* produces distorted sex ratios in some crosses (Roco *et al.* 2015). Thus, African clawed frogs use at least two systems for sex determination, and at least one of them evolved during the diversification of this group.

Within subgenus *Xenopus*, species in a clade including *X. borealis* (Parker 1936), *X. muelleri* (Peters 1844), and *X. fischbergi* (Evans *et al.* 2015) appear to lack *DM-W* (Bewick *et al.* 2011), hinting at additional diversity of sex chromosomes in this group. The phylogenetic placement of this clade within *Xenopus* remains uncertain, making unclear the evolutionary histories of potentially diverse triggers for sex determination.

To further explore sex-related innovations in these frogs, we (i) used whole transcriptome information from several species to further resolve phylogenetic relationships within subgenus *Xenopus*. We (ii) tested whether *DM-W* is sex-linked in the most distantly related species from *X. laevis* that is known to carry *DM-W*, *i.e.*, *X. clivii* (Peracca 1898). Then, we (iii) used reduced representation genome sequencing and Sanger sequencing to identify the sex-linked region in *X. borealis*, and (iv) established homology between the genes on the sex chromosomes of *X. borealis* and several other distantly related species. Our results identify a new sex determination system in *X. borealis* that evolved after the *DM-W*-based system was already in place in an ancestor. Interestingly, the genomic regions involved in sex determination of *X. borealis* and therian mammals (including humans) are homologous. Rapid evolution of *Xenopus* sex chromosomes highlights a central role for cooption of genes with conserved developmental roles in the evolution of important genetic pathways.

## Materials and Methods

### Exploring the origin of DM-W

#### Nuclear data:

In order to infer evolutionary relationships among representative *Xenopus* species that do and do not carry *DM-W*, we performed phylogenetic analyses on nuclear sequence data obtained from two sources. For the tetraploid species *X. laevis* and the diploid outgroup species *X. tropicalis*, we used Unigene databases (downloaded November 2015). These datasets had 31,306 and 36,839 unique sequences for *X. laevis* and *X. tropicalis*, respectively. For the tetraploid species *X. borealis*, *X. clivii*, *X. allofraseri*, and *X. largeni*, we extracted RNA from liver tissue using the RNAEasy extraction kit (Qiagen Inc.). These four transcriptomes were multiplexed on two thirds of one lane of an Illumina HiSeq 2000 machine, with 100 bp paired end sequencing and using libraries that were prepared with the Illumina TruSeq RNA Sample Preparation Kit v2. This produced 18–20 million paired reads for each sample (data are deposited in the NCBI short read archive with accession numbers: *X. borealis* PRJNA318484, *X. clivii* PRJNA318394, *X. allofraseri* PRJNA318474, and *X. largeni* PRJNA318404).

Low quality reads and bases were removed using Trimmomatic version 0.30 (Bolger *et al.* 2014). We discarded the first and last 3 bp and then required the average Phred-scaled quality scores of retained sequences to be at least 15 in a sliding window of 4 bp. After imposing these requirements, we discarded all reads that were shorter than 36 bp. Across the samples, 88–95% of paired reads passed these filters. We then assembled the transcriptomes for each species with Trinity (version 2013_08_14), using default values for all settings including, for example, a kmer size of 25 and a minimum contig length of 200 (Grabherr *et al.* 2011; Haas *et al.* 2013). The resulting assemblies had 72,000–97,000 unique transcripts (*X. borealis* = 81,696, *X. clivii* = 72,019, *X. allofraseri* = 96,832, and *X. largeni* = 82,695) and N50 values (the minimum length, in bp, for the longest 50% of reads) ranging from 885–1176 bp (*X. borealis* = 1078, *X. clivii* = 885, *X. allofraseri* = 1176, and *X. largeni* = 1000). Additional information on Illumina sequencing is presented in Supplemental Material, Table S1.

We used a reciprocal BLAST (Altschul *et al.* 1997) approach between each tetraploid transcriptome (or Unigene database in the case of *X. laevis*) and the *X. tropicalis* Unigene database to collect sets of homologous sequences for phylogenetic analysis (Figure S1). These sets of sequences included orthologous gene sequences (sequences in different species whose divergence was triggered by speciation), homeologous gene sequences (sequences in the same or different species whose divergence was triggered by genome duplication), and included splice variants, segmental duplicates, and assembly errors generated by Trinity (Grabherr *et al.* 2011). We performed a quality control step, retaining only those alignments whose ungapped length was above an arbitrary cutoff of 299 bps, and that contained sequences from at least three ingroup species with at least one species having at least two sequences. The need for the requirement that at least one species have two (possibly homeologous) sequences is discussed next.

Because our ingroup species are tetraploid, it was crucial for our phylogenetic analyses to distinguish orthologous from homeologous gene sequences. Since speciation occurred more recently than whole genome duplication in subgenus *Xenopus*, orthologous genes are expected to be more closely related to one another than they are to homeologous genes. In a gene tree with only one sequence from each species, it was therefore a concern that the relationships among the sequences could be orthologous or homeologous. Therefore, we developed a phylogeny-based bioinformatic filter that identified alignments whose estimated phylogeny allowed us to distinguish orthologous from homeologous gene sequences (Figure S1). Importantly, we did not make any assumptions about how the orthologous sequences were related to one another. This filter involved three rounds of tree building, with each followed by assessment of sequence relationships using a script and functions from the R packages Ape, Phytools, and Phangorn (R Core Team 2015; Revell 2012; Schliep 2011; Paradis *et al.* 2004; this script is available at Dryad repository; see *Data availability*). The resulting alignments each included at least one species with two homeologous sequences, which diverged prior to speciation of extant tetraploids in subgenus *Xenopus*. Additionally, each alignment had at least three representative orthologous sequences. Similar BLAST and phylogenetic-based filtering approaches have been used in other studies to distinguish orthologous from homeologous gene sequences (Dehal and Boore 2005; Inoue *et al.* 2015). See File S1, section S1.1 for full details.

#### Phylogenetic analyses of nuclear DNA:

After filtering these alignments, we performed several phylogenetic analyses on these data including: (i) individual gene tree analyses for each alignment (Beast; Drummond and Rambaut 2007), (ii) concatenated Bayesian analyses (Beast), (iii) concatenated maximum likelihood analyses (RAxML; Stamatakis 2014), (iv) a gene tree to species tree analysis using MPest (Liu *et al.* 2010), and (v) a multi-species coalescent analysis using *Beast (Heled and Drummond 2010). For Analysis (i), a model of evolution was selected for each gene alignment using the Akaike Information Criterion MrModelTest2 (Nylander 2004). We set the root height to be 65 MY, with a SD of 4.62 MY (Bewick *et al.* 2012) and assumed a strict clock, and ran two chains, for at least 75 million generations. 197 files failed to converge with substitution model selected by MrModelTest, so we instead used the HKY +Γ model. For all analyses, we assessed convergence of the posterior distribution using loganalyser (part of the Beast package), and removed a 25% burn-in from each chain. For Analysis (i), we summarized relationships across the combined postburn-in posterior distribution of all individual gene analyses using an approach described in File S1, section S2.2. We analyzed two datasets for Analyses (ii) and for (iii). The first dataset was a concatenation of all gene alignments. The second dataset had all sites with gaps or missing data removed from the concatenated alignment. For Analysis (ii), for both datasets, we set a GTR + I + Γ substitution model (as selected by MrModelTest using AIC) and a strict clock with an exponential distribution for the rate with a mean rate of 1.0 and a SD of 0.33 (default settings in Beauti). The root height was set to 65 my (±4.62) as detailed above (Bewick *et al.* 2012). For each dataset, we ran four independent chains, for 50 million generations, and tested for convergence by inspecting the plots of parameter estimates and calculating ESS values using Tracer. Based on this inspection, we removed a 25% burn-in from each chain and constructed a consensus tree using TreeAnnotator. For Analysis (iii), we used the GTR + Γ model and performed 500 bootstrap replicates to assess support.

For Analysis (iv), we used the individually constructed Beast consensus chronograms that were generated from Analysis (i). We selected a random sample of 250 trees from the postburn-in posterior sample of tree topologies from each gene tree analysis to act as the “bootstrap” replicates, which MPest uses to assess support (Seo 2008). These trees were uploaded to the Straw server (Shaw *et al.* 2013) to run the MPest analysis.

To perform Analysis (v), we used only those gene alignments that had orthologous sequence data for all species (*i.e.*, five aligned orthologs within one homeologous lineage), and retained only the longest sequence in the other homeologous lineage (or a randomly selected sequence if there were multiple equally long sequences). Because the homeologous sequences are equivalently diverged from a set of orthologs, it did not matter from which species this latter homeologous sequence was derived. The result was a dataset that had gene sequence for all taxa, and minimizing missing data to only incomplete sequencing of a gene and insertion deletion mutations. We ran *Beast with a strict clock that was linked across all partitions. The GTR + Γ model of evolution was used and was linked across partitions. The tree topology, however, was free to vary among genes (*i.e.*, it was unlinked). We ran two independent chains for 500 million generations each. Convergence was assessed using effective sample size values calculated with Tracer. Based on this, we removed a 25% burn-in from each chain. This analysis did not include calibration points because all attempts to set one failed to converge on the posterior distribution. Instead, in order to assign dates to the nodes, trees in the resulting posterior distribution were rescaled using an *R* script that used functions from the phytools library (Revell 2012). As above, the root node age was drawn from a normal distribution with a mean of 65 and a SD of 4.62 (Bewick *et al.* 2012), and the rest of the nodes were assigned based on branch length from the root.

#### Phylogenetic analysis of mitochondrial DNA:

We downloaded the previously sequenced mitochondrial genomes for *X. tropicalis* (direct GenBank submission: NC_006839.1), *X. borealis* (GenBank accession no. X155859; Lloyd *et al.* 2012), and *X. laevis* (GenBank accession no. HM991335; Irisarri *et al.* 2011). We used the *X. borealis* mitochondrial genome as a Blast query to recover matches from the transcriptomes of *X. clivii*, *X. allofraseri*, and *X. largeni*, retaining hits with less than an *e*^−10^ match. Then, using these assembled mitochondrial DNA (mtDNA) sequences and the previously sequenced mtDNA genomes, a multispecies alignment was performed using Mafft (Katoh and Standley 2013) followed by manual adjustment. In order to remove sections that were poorly aligned or had ambiguous homology, GBlocks (Castresana 2000) was used with default parameters. We then performed a Beast analysis of these data, a root node age set to 65 MY and a SD of 4.62 (Bewick *et al.* 2012), a GTR + I + Γ substitution model (as determined by AIC with MrModelTest2), and ran 13 chains. For comparative purposes, we ran this analysis with a relaxed clock and with a strict clock, and the suitability of each clock model was assessed by comparing the harmonic means of the postburn-in likelihood values. We also performed a RAxML analysis with a GTR + Γ model and 1000 bootstrap replicates to assess support.

### Assessing sex specificity of DM-W in X. clivii

The phylogenetic results (discussed below in DM-W originated before speciation of *X*. *laevis*, *X*. *clivii*, *X*. *borealis*, and other 4x = 36 tetraploids) suggests that *X. clivii* is the most distantly related species to *X. laevis* that carries *DM-W*. Therefore, we tested whether *DM-W* is found only in *X. clivii* females by attempting to amplify *DM-W* in several wild-caught individuals for which sex was inferred based on external morphology (Evans *et al.* 2011a). We designed primers from a sequenced clone of *DM-W* from this species (Bewick *et al.* 2011; Table S2) and attempted to amplify this gene in 12 females and 13 males.

### The sex determining region of X. borealis

#### X. borealis and X. laevis families:

We generated *X. borealis* and *X. laevis* families from adults obtained from *Xenopus* Express (Brooksville, FL). To promote mating, parents each received 50 U of Human chorionic gonadotropic (HCG) followed by 200 and 50 U for the female and male, respectively, 6 hr later. The *X. borealis* offspring were reared to sexual maturity, killed with an overdose of MS222, and dissected to determine sex based on presence of testis or ovary. For *X. laevis*, tadpoles were reared for 4 wk and then killed with MS222. Sex of the *X. laevis* tadpoles was determined based on amplification or lack of amplification of a portion of *DM-W*; amplification of *DMRT-1* was used as a positive control (Yoshimoto *et al.* 2008; Bewick *et al.* 2011). For both families, DNA was extracted using DNEasy kits (Qiagen, Inc.) from either fresh liver tissue (*X. borealis*) or tadpole tail tissue (*X. laevis*).

#### Genotype by sequencing (GBS) sequencing:

To identify the sex determining region of *X. borealis*, we performed GBS (Elshire *et al.* 2011) on parents and offspring of the *X. borealis* cross. DNA was extracted for 23 male and 24 female siblings, and both parents using DNEasy extraction kits (Qiagen, Inc.). For the mother and father, we sequenced multiple technical replicates to increase coverage 10-fold for each parent compared to each offspring. Library preparation using the *Eco*T22I restriction enzyme and sequencing was performed at Cornell University Institute of Biotechnology Genome Diversity Facility. Sequencing (100 bp, single end) was performed using an Illumina Hi-Sequation 2500 machine; 96 samples, of which 67 were *X. borealis* samples for this study, were repeated on two Illumina lanes at 96-plex each; the resulting sequence files were merged prior to processing.

We then used Tassel v.3.0 (Glaubitz *et al.* 2014), employing the UNEAK pipeline (Lu *et al.* 2013), to perform SNP calling of GBS data without the use of a reference genome sequence. Tassel also does demultiplexing, quality checking, and barcode trimming of sequences. During the process, reads were truncated to a maximum of 64 bp, and high quality reads with < 64 bp were padded with “A” nucleotides to bring them to the 64 bp length. We set the minimum number of times a read must be present (-c option) to five, and set the error tolerance rate (*i.e.*, the number of mismatched base pairs between reads) to 0.03 when forming groups of homologous sequences. The minimum and maximum allele frequencies of SNPs were set to 0.05 and 0.5, respectively, and the minimum and maximum call rate (*i.e.*, the proportion of all individuals that must have a sequence to call a SNP for a stack of reads) was set to 0.0 and 1.0, respectively. We then trimmed the dataset to only sequence tags that had SNP calls for at least 90% of individuals.

One concern we encountered was “under calling” of heterozygous sites, wherein sites that are actually heterozygous were called as homozygous. For instance, if the parental genotype calls were A/T and A/A, and an offspring was T/T, then it is likely the offspring was actually T/A because the coverage of the parents was ∼10 × higher. To cope with this, we used a Perl script (deposited in Dryad, see above) to compare offspring genotype calls to those of parent genotype calls for each locus in order to identify biologically implausible genotypes. If < 10% of offspring had a biologically implausible genotype call, then the implausible genotype calls were changed to missing genotypes. If more than 10% of the offspring had implausible calls, then the site was discarded. With this Perl script, we then identified completely sex biased inheritance of parental SNPs, and used this information to determine whether such sites had inheritance consistent with a female heterogametic (ZZ/ZW) or male heterogametic (XX/XY) sex determining system. We limited our search to loci that were completely sex biased (*i.e.*, only daughters or only sons were heterozygous).

#### Comparative analysis of the X. borealis sex determining region:

We used Blast with the consensus sequences (64 bp long) surrounding the sex-linked SNPs (hereafter “tags”) from *X. borealis*, generated by Tassel, as a query to find matches in the *X. laevis* genome assembly v.7.1 (Bowes *et al.* 2008). Matching *X. laevis* scaffolds were then aligned to the reconstructed *X. tropicalis* chromosomes in the v.9.0 genome, using the program Nucmer (part of the Mummer package; Delcher *et al.* 2002). Settings for Nucmer included a minimum length of a maximal exact match of 50 (-l 50), gaps between cluster of matching sequence was set to 500 (-g 500), match separation was set at 0.08 (-d 0.08), and the minimum cluster length was set to 150 (-c 150).

As discussed below, this analysis indicated that a genomic region containing three sex-related genes—*sex determining region Y-box 3* (*SOX3*), *androgen receptor* (*AR*), and *fragile X mental retardation 1* (*FMR1*)—might be sex-linked in *X. borealis*. To test this, we amplified and sequenced portions of these three genes in our *X. borealis* family using Sanger sequencing. Primers for both homeologs of *SOX3* and *FMR1* were designed from the *X. laevis* v.7.1 genome or from unpublished *X. borealis* genome sequence data. For *AR*, we used the primers detailed in Evans *et al.* (1998), which target the hypervariable region of one homeolog of the *AR* gene. Primer sequences are reported in Table S2. Using BLAST, we identified chromosomes or scaffolds in the *X. laevis* genome v.9 that are orthologous to these homeologous sequences in *X. borealis*. We also sequenced these genes in wild-caught *X. borealis*, including individuals of both sexes and from multiple localities.

As an additional independent test of whether the sex-determining regions of *X. laevis* and *X. borealis* reside in nonhomologous genomic regions, we evaluated sex linkage of a *RAB6A* homeolog that is located near *DM-W* (Uno *et al.* 2013), in both the *X. laevis* and *X. borealis* families. We designed primers for both homeologs using *X. laevis* genome v.7.1 (Table S2) and amplified in parents and offspring of both crosses, followed by Sanger sequencing.

### Data availability

Representative individuals from the sex-linked alignments and wild samples were deposited in Genbank (accession *SOX3*:KX765742–KX765751; *FMR1*:KX765752–KX765762; and *AR*:KX765731–KX765741), and transcriptome and GBS sequences in the NCBI short read archive (accessions PRJNA318484, PRJNA318394, PRJNA318474, PRJNA318404, and PRJNA319044). The phylogenetic trees, gene sequence alignments, Beast XML files for final gene trees, important scripts used in this study, and full alignments of sex-linked genes are deposited in Dryad (doi: 10.5061/dryad.00db7).

## Results and Discussion

### DM-W originated before speciation of X. laevis, X. clivii, X. borealis, and other 4x = 36 tetraploids

The gene *DM-W* triggers female sexual differentiation in the African clawed frog *X. laevis* and is located on the female-specific portion of the W sex chromosome (Yoshimoto *et al.* 2008). This gene is carried by several other *Xenopus* species, but has not been detected in *X. borealis* (Bewick *et al.* 2011). The most distantly related species from *X. laevis* known to carry *DM-W* is *X. clivii*; however, phylogenetic relationships among these three species remain unresolved. If *X. borealis* does indeed lack *DM-W*, two possibilities exist: either (i) *DM-W* arose after divergence of *X. borealis* from the most recent common ancestor (MRCA) of species that carry this gene, including *X. laevis* and *X. clivii*, or (ii) *DM-W* evolved prior to this in the MRCA of species that do and do not carry *DM-W*, and was subsequently lost in a more recent ancestor of *X. borealis*. Analyses of partial mtDNA sequences support the former hypothesis (Evans *et al.* 2004, 2011b, 2015) and analysis of two linked nuclear DNA (nDNA) genes supports the latter (Evans *et al.* 2005, 2015; Evans 2007). Therefore, we estimated phylogenetic relationships among tetraploid species that represent the major *Xenopus* clades, in which *DM-W* has and has not been detected, using new and publicly available sequence data from nuclear and mitochondrial DNA from *X. largeni*, *X. allofraseri*, *X. borealis*, *X. clivii*, and *X. laevis*, and the diploid outgroup species *X. tropicalis*.

From these data, we recovered 1585 sets of homologous nuclear gene sequences (File S1, section S1.1). Each set consisted of at least one species with two homeologous sequences (*i.e.*, generated from tetraploidization), at least 300 bp for all species, and a minimum of three ingroup taxa for at least one set of orthologs. When combined, these data included 2,696,030 bp. Data from a given ingroup species were missing from the gene alignments as rarely as 14% of the gene alignments (for *X. laevis*) to as much as 64% of the gene alignments (for *X. clivii*, File S1, section S1.1). These data formed the basis of Analyses (i–iv). Analyses with gapped sites removed [alternate Analysis (ii) and (iii)], included a total of 788,627 aligned bp. The *Beast analysis [Analysis (v)] included 151 gene alignments (238,606 bp, 70,233 sites sequenced for all taxa, with some gaps due to insertion-deletion mutations or incomplete gene sequences).

**Figure 1:**
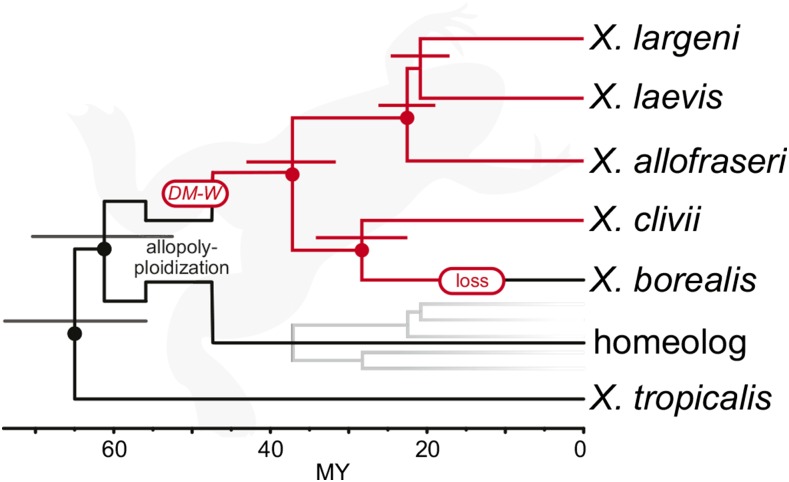
Phylogenetic relationships inferred from representative species in subgenus *Xenopus* suggests *DM-W* was gained before diversification of (4*x* = 36) tetraploids and then lost in an ancestor of *X. borealis*. This phylogeny was recovered from *Beast analysis of transcriptome data and is topologically consistent with those recovered from other analyses of nuclear DNA and of mitochondrial DNA. Dots over nodes indicate 1.0 posterior probability; bars above nodes indicate the 95% credible intervals for divergence time in millions of years (MY). All species depicted are tetraploids except the outgroup species, *X. tropicalis*, which is diploid. For this analysis, one homeolog from any one of the tetraploid species was included for each gene, and is indicated by the gray subtree (File S1, section S1.1). The timing of the origin of *DM-W* with respect to the allopolyploidization event (whether before or after) is unclear. *Xenopus* silhouette from Phylopic by Sarah Werning, CC04 license.

All of the multigene analyses (ii–v) strongly supported, with posterior probabilities of 1.0 (or bootstrap support of 100%), two reciprocally monophyletic clades, with the first including *X. borealis* and *X. clivii* and the second including *X. laevis*, *X. largeni*, and *X. allofraseri* (Figure 1, Figure S2, and File S1, section S2.1). Similar to previous studies (Evans *et al.* 2004, 2005, 2007, 2011b, 2015), these analyses failed to resolve relationships among *X. laevis*, *X. largeni*, and *X. allofraseri* with strong support (Figure 1, Figure S2, and File S1, section S2.1). Analyses of individual genes (i) identified substantial gene tree discordance among chronograms estimated from each gene (Table S3 and File S1, section S2.2). Despite this discordance, in the pooled postburn-in posterior distribution of these chronograms, a sister relationship between *X. borealis* and *X. clivii* was at least twice as common as any other relationship with either of these species (Table S3 and File S1, section S2.2).

Because previous phylogenetic inferences from mtDNA and nDNA differed with respect to the placement of *X. clivii*, we reexamined mtDNA relationships with additional data from the liver transcriptome sequences of *X. clivii*, *X. largeni*, and *X. allofraseri*, and complete mtDNA genome sequences from *X. tropicalis*, *X. laevis*, and *X. borealis*. After gaps and ambiguously aligned portions were removed, the alignment length was 8318 bp, which spans about 50% of the complete mtDNA genomes of *X. laevis*, *X. borealis*, and *X. tropicalis*. When analyzed with a relaxed molecular clock Bayesian analysis, or with a no clock maximum likelihood analysis, a phylogeny that was topologically consistent with the nDNA analyses was recovered. This topology included a clade containing *X. borealis* and *X. clivii*, although support for this clade was lower than the multigene analyses of nDNA described above (posterior probability was 0.75 and bootstrap support was 66%; Figure S3). Analysis with manual removal of ambiguously aligned sequences instead of Gblocks (16,260 bp aligned) recovered the same topology for both analyses and with similar levels of support (results not shown).

Analysis with a strict molecular clock supported an alternative mtDNA topology, with *DM-W* containing species forming a monophyletic group, as was found by previous studies (Evans *et al.* 2004, 2015). However, Bayes factors calculated following Nylander *et al.* (2004), indicate that a relaxed clock model is strongly preferred over the strict clock (BF = 9.3; Kass and Raftery 1995). An important difference between this and previous mtDNA analyses is that this study is based on a ∼sixfold larger dataset. Similar to the nDNA analyses, mtDNA analyses failed to confidently resolve the relationships of *X. laevis*, *X. allofraseri*, and *X. largeni* (Figure S3).

Although the support for a sister relationship of *X. borealis* and *X. clivii* is lower in the mtDNA analysis than in the nDNA analyses, this relationship has more support than any alternative. Thus, using the most favored models of evolution we considered, the most strongly supported phylogenetic relationships among nDNA and among mitochondrial DNA are both consistent with an origin of *DM-W* prior to the diversification of the most recent common ancestor of all of our ingroup taxa (*X. laevis*, *X. largeni*, *X. allofraseri*, *X. clivii*, and *X. borealis*). Results from mtDNA and nDNA, thus, both suggest that *DM-W* originated before the diversification of extant (4*x* = 36) tetraploids in subgenus *Xenopus*.

### DM-W is sex-linked in X. clivii

Our phylogenetic results indicate that *X. clivii*, a species that carries *DM-W*, is closely related to several species in which *DM-W* has not been detected, including *X. borealis* (Bewick *et al.* 2011). *DM-W* was previously amplified in one female *X. clivii* individual, but it is not clear whether this gene is also sex-linked in this species. Put another way, although *DM-W* arose before *X. laevis* and *X. clivii* diverged from one another, it is possible that *DM-W* acquired its role as a trigger for sexual differentiation (and thus its female-specific mode of inheritance) in an ancestor of *X. laevis* after divergence from an ancestor of *X. clivii*. Therefore, we tested whether *DM-W* is found only in *X. clivii* females, including in our assay males and females from the populations on each side of the Ethiopian Rift Valley (Evans *et al.* 2011a). We were able to amplify *DM-W* in a subset of females (8 of 12 females) from both sides of the Rift Valley, but no males (0 out of 12 males; a 13th male also failed to amplify in a positive control; Figure S5). The failure of *DM-W* to amplify in four female samples, which were also from both sides of the Ethiopian Rift Valley, could be due to divergence at our primer sites or misidentification of the sex of these individuals when sampled in the field (specimens of these individuals were not available for examination). It is also possible that additional sex determining systems may also be present in *X. clivii*, as is the case in *X. tropicalis* (Roco *et al.* 2015). Either way, female-specific amplification is consistent with the hypothesis that *DM-W* is found only in female *X. clivii*, that this gene triggers female sexual differentiation in at least some *X. clivii* individuals, and (more broadly) that *DM-W* was the ancestral trigger for female differentiation in subgenus *Xenopus*.

### The sex determining region of X. borealis is different from that of X. laevis and that of X. tropicalis

Our inability to detect *DM-W* in *X. borealis* could be because this gene is not present, or because divergence at primer sites prevented amplification with the polymerase chain reaction. To find the sex-linked region of *X. borealis*, we examined patterns of inheritance of SNPs identified in our GBS data from the *X. borealis* family. Of the ∼89,000 SNPs identified by Tassel (Table S1), ∼21,000 were successfully genotyped in at least 90% of the offspring, and 15,632 of these passed our filter because they had “undercalled” genotypes in < 10% of the offspring (*Materials and Methods*). Of these, variation in 25 SNPs had a completely sex-linked pattern of inheritance (in offspring one sex is completely homozygous and the other completely heterozygous). By inspecting the genotypes of the parents, we could then distinguish ZZ/ZW from XX/XY systems (Figure S4). All 25 tags were consistent with female heterogamy. In 24 of them, the mother and daughters were heterozygous and the father and sons were homozygous; a pattern best explained by a SNP on the W chromosome. In one of the 25 tags, the mother and sons were heterozygous and the father and daughters were homozygous; a pattern consistent with a SNP on the Z chromosome of the mother that was not present in either Z chromosome of the father. Overall, these results support genetic sex determination and female heterogamy in *X. borealis*, at least in the strain we examined, which is also the case in *X. laevis* (Mikamo and Witschi 1966) and possibly all other *DM-W*-containing *Xenopus* species.

To evaluate homology of the sex determining regions of *X. borealis*, *X. laevis*, and *X. tropicalis*, we aligned the *X. borealis* tags to the *X. laevis* genome assembly. This resulted in tags matching either (i) one region in *X. laevis*, (ii) two regions, (iii) multiple regions, or (iv) no regions. Scenario (ii) is likely the result of the short tags matching both homeologs in the *X. laevis* genome with similar strength. Scenarios (iii) and (iv) are not surprising given the short length of the tags and the divergence between *X. laevis* and *X. borealis* (Figure 1), and we discarded these tags. Ten of the 25 tags had only one or two *X. laevis* scaffold matches below our BLAST threshold (<*e*^−5^). Six of these 10 scaffolds (either the single match or a randomly retained scaffold if there were two matches) aligned to *X. tropicalis* chromosome XTR8, two scaffolds had a split alignment with portions of each matching two different *X. tropicalis* chromosomes (XTR1 and XTR5 or XTR3 and XTR6, respectively), one matched *X. tropicalis* chromosome XTR4, and one matched *X. tropicalis* chromosome XTR7.

Most of the tags mapped to the XTR8 chromosome, suggesting that the sex chromosomes in *X. borealis* might be homologous to this *X. tropicalis* chromosome. To test this, we designed homeolog-specific primers based on *X. laevis* sequences, to amplify and sequence three genes (*SOX3*, *AR*, and *FMR1*) in our *X. borealis* family that are known to reside on chromosome XTR8 in *X. tropicalis* (Uno *et al.* 2013). This effort identified sex-linked polymorphisms in *X. borealis* in one homeolog of each gene, and each was consistent with a female heterogametic (ZZ/ZW) sex chromosome system. For *SOX3*, *AR*, and *FMR1*, we successfully amplified and genotyped 93, 41, and 54 offspring, respectively, including 47, 24, and 30 daughters, respectively. For all three of these genes, we identified at least one heterozygous site in the mother of the cross that allowed us to confirm sex linkage and female heterogamy (Figure S4; alignments of all sequences are deposited in Dryad and representative sequences are deposited in GenBank; see *Data availability*). For the *AR* amplification, the father appeared to have a null allele, but importantly, this did not compromise our ability to assess sex linkage and female heterogamy, which was based on patterns of inheritance of a heterozygous SNP from the mother (Figure S4), resulting in completely sex associated genotypes in the offspring. The top BLAST hit of the sex-linked *X. borealis SOX3* and *FMR1* homeologs to the *X. laevis* genome indicated that these sequences were orthologous to *X. laevis* chromosome XLA8L (and thus homeologous to XLA8S); *AR* was orthologous to an unplaced scaffold (scaffold 37), but fluorescent *in situ* hybridization studies place this gene on XLA8L (Uno *et al.* 2013).

In wild-caught *X. borealis*, we successfully sequenced amplifications from three females and three males for *SOX3*, and amplifications from the same individuals plus a fourth male for *AR* and *FMR1*. Two of three females tested had the same heterozygous genotypes in *SOX3* and *FMR1* as the females in our lab family; for *AR*, neither of these samples had the same sex-linked polymorphism as the lab family. The wild-caught females also had other polymorphic sites, some of which were shared with male wild samples. These results indicate either that these genes reside in the pseudoautosomal region in *X. borealis*, that there is variation in the sex determining system within *X. borealis*, or some combination of these possibilities. It is also possible that the sex of some of the wild-caught individuals was misidentified based on external morphology; unfortunately, specimens of these individuals were not available for examination. Examination of other wild-caught individuals whose sex is determined surgically is an important next step for further characterizing the sex-specific region of the sex chromosomes of *X. borealis*.

Analysis of polymorphisms in Sanger sequences of homeologs of the *RAB6A* gene indicated that one homeolog is linked to *DM-W* in *X. laevis*, as indicated by sex-linked inheritance (File S1, section S2.3), in agreement with a finding from fluorescence *in situ* hybridization (Uno *et al.* 2013). This analysis also revealed that the ortholog of *RAB6A* that is sex-linked in *X. laevis* is not sex-linked in *X. borealis* (File S1, section S2.3). Overall, these results demonstrate that the genomic region containing the trigger for sex determination differs between the *X. borealis* strain we examined and *X. laevis*.

### Some genomic regions are good at sex determination

**Figure 2:**
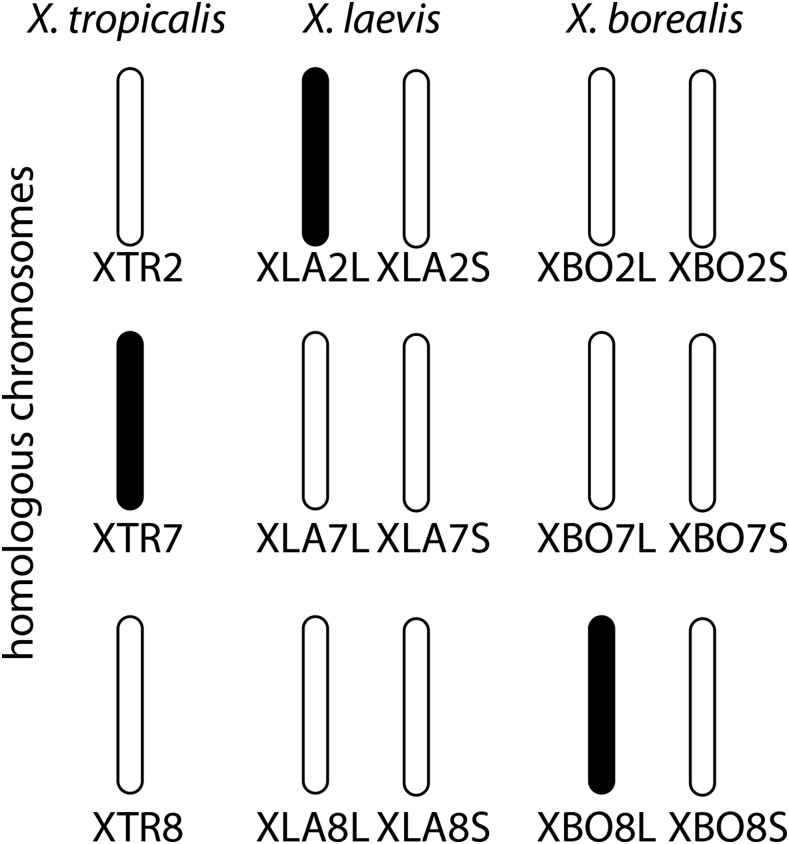
Sex chromosomes, indicated in black, in three species of African clawed frog are not homologous. For the tetraploid species, *X. laevis* and *X. borealis*, both homeologous (L and S) chromosomes are shown. Chromosome nomenclature for *X. tropicalis* and *X. laevis* follows (Matsuda *et al.* 2015).

Our results indicate that the sex chromosomes of *X. borealis* are homologous to *X. tropicalis* chromosome XTR8, orthologous to *X. laevis* chromosome XLA8L, and homeologous to *X. laevis* chromosome XLA8S (Figure 2). In the diploid species *X. tropicalis*, the gene that triggers sex determination is unknown, but resides on the distal end of the petite arm of chromosome XTR7 (Olmstead *et al.* 2010; Wells *et al.* 2011; Roco *et al.* 2015). XTR7 is homologous to *X. laevis* autosomes XLA7L and XLA7S (Matsuda *et al.* 2015; Uno *et al.* 2013). The sex chromosome of *X. laevis* is XLA2L; this chromosome and its homeologous chromosome XLA2S are homologous to XTR2 of *X. tropicalis* (Matsuda *et al.* 2015; Uno *et al.* 2013). Thus, at least three sets of nonhomologous sex chromosomes are present within the African clawed frogs (Figure 2). The sex chromosomes of *X. laevis* and *X. borealis* occur in orthologous subgenomes (*i.e.*, portions of their respective allotetraploid genomes that are derived from the same diploid ancestor). There is still the possibility that *DM-W* has been translocated to the newer sex chromosomes in *X. borealis*, but all efforts to detected it have failed (Bewick *et al.* 2011 and here).

Another frog species, *R. rugosa*, has sex chromosomes that are at least partially homologous to those in *X. borealis* (and this may be true for two other *Rana* species; Miura 2008). In both species, *SOX3* and *AR* are located on the sex chromosomes (Fujii *et al.* 2014; Uno *et al.* 2015). Interestingly, this inference extends even farther: orthologs of *AR*, *SOX3*, and *FMR1* are also present on the X chromosome of therian mammals, including humans (Uno *et al.* 2013), and *SOX3* is a new trigger for sex determination in a fish (*O. dancena*; Takehana *et al.* 2014). Similarly, the Z chromosome of lacertid lizards is partially homologous to the X chromosomes of therian mammals (Rovatsos *et al.* 2016). The phylogenetic placement of these lineages with respect to other species that have different sex determining systems [specifically *X. laevis* and monotremes (Veyrunes *et al.* 2008)] strongly suggests several independent origins of sex linkage of these homologous regions, or minimally of regions containing *SOX3*. Similarly, another region containing *DMRT-1*, an ortholog of which is related by partial gene duplication (paralogous) to *DM-W*, independently became sex-linked in birds and a gecko lizard (Kawai *et al.* 2009). Taken together, these observations are consistent with the proposal that certain genomic regions contain blocks of genes that are particularly suited to perform the task of triggering sex determination (Graves and Peichel 2010; Brelsford *et al.* 2013).

### Conclusions

Sex chromosomes carry the genetic trigger that initiates sexual differentiation, a crucial developmental phenomenon that is generally required for reproduction (Matzuk and Lamb 2008). Sex chromosome turnover could occur by translocation among chromosomes of a conserved genetic trigger, or via a novel mutation creating a new trigger on an autosome. That sex chromosomes in African clawed frogs and several other lineages have frequently turned over contrasts sharply with other lineages with ancient sex chromosomes, such as therian mammals. Indeed, transitions in sex chromosomes appear to be more frequent when sex chromosomes are cytologically homomorphic and/or nondifferentiated (Bachtrog *et al.* 2014), which is the case in *Xenopus*, including *X. borealis* (Tymowska 1991), but not therian mammals. However, the evolutionary dynamics of these systems are highlighted by loss of the Y chromosome in various therians (Just *et al.* 1995; Sutou *et al.* 2001) and duplication of *SRY*, an ancient trigger for sex determination in this group (Geraldes *et al.* 2010).

A lack of recombination in the genomic region carrying the trigger for sex determination causes sex chromosomes to diverge from one another (Rice 1987). If the region of suppressed recombination expands, as it did in therian mammals, genomic elaborations such as loss and dosage compensation of sex-linked genes may arise and act as “evolutionary traps” that impede evolutionary change or, more specifically, future sex chromosome turnover (Bull 1983; Pokorna and Kratochvíl 2009; Gamble *et al.* 2015). In theory, before such evolutionary traps evolve, genes with sexually antagonistic function could catalyze sex chromosome turnover by increasing the fixation probability of new sex-determining genes that arise on a linked autosomal region (van Doorn and Kirkpatrick 2007). Related to this, dosage compensation has not been detected in species with female heterogamy (Mank 2009; Vicoso and Bachtrog 2009) or in anurans (frogs) in general, (*e.g.*, Schmid *et al.* 1986), and is unlikely to exist in *Xenopus* species whose female heterogametic sex chromosomes are homomorphic at the cytological (Tymowska 1991) and molecular level (Bewick *et al.* 2013). An absence of dosage compensation may prevent sex chromosome divergence (Adolfsson and Ellegren 2013) leaving a permissive environment for sex chromosome turnover in the presence of maintained homomorphic sex chromosomes, thereby avoiding these evolutionary traps. However, this is not always the case, as some snakes have differentiated sex chromosomes despite a lack of global dosage compensation (Vicoso *et al.* 2013; Rovatsos *et al.* 2015). More information about the nature of the master trigger for sex determination in *X. borealis*, on sex-linked genes, and on sex-biased expression of genes elsewhere in the genome may cast additional light on the drivers of sex chromosome turnover in these frogs. The drivers could include the role of alternative mechanisms that could resolve sexual conflict, such as gene duplication (Gallach *et al.* 2011; Wyman *et al.* 2012), which is a potentially important factor in these tetraploid species.

The sex determination system we detected in *X. borealis* is set apart from most other rapidly evolving systems, in that it is derived from an ancestral trigger that itself was newly evolved (*i.e.*, *DM-W*), as opposed to groups with diverse mechanisms that are each potentially once-evolved (autapomorphic). Our results support the hypothesis that *X. borealis* and *X. clivii* are sister taxa, and that *DM-W* is restricted to female *X. clivii*. This suggests that female sexual differentiation was triggered by *DM-W* in the ancestor of all extant species of subgenus *Xenopus*. This also suggests that the new system we report in *X. borealis* is derived with respect to the *DM-W*-based system. Thus, the sex chromosomes of *Xenopus* are an example of multiple important biological novelties arising in rapid succession.

Perhaps most interesting, however, are the aspects of sex determination that convergently evolve in distantly related organisms in the context of frequent sex chromosome turnover. These aspects include the participation of key sex-related genes (*e.g.*, *DMRT-1* in *X. laevis* and in *O. latipes*) and the role of homologous genomic regions (*e.g.*, carrying *SOX3*, in *X. borealis* and in *O. dancena*). This study contributes to a growing body of evidence that, in lineages with rapidly changing sex chromosomes, the turnover is catalyzed by cooption of genetic building blocks that are already involved in the development and maintenance of sexual differentiation.

## 

## Supplementary Material

Supplemental Material
