# Supplementary material for: Sequential Turnovers of Sex Chromosomes in African Clawed Frogs (Xenopus) Suggest Some Genomic Regions Are Good at Sex Determination
Source: G3 (Bethesda). 2016 Sep 7;6(11):3625–33. doi: 10.1534/g3.116.033423 (PMC5100861; doi:10.1534/g3.116.033423)
Supplement: Supplemental Material [file supp_6_11_3625__index.html]

Sequential Turnovers of Sex Chromosomes in African Clawed Frogs (Xenopus) Suggest Some Genomic Regions Are Good at Sex Determination — Supplemental Material 

# Sequential Turnovers of Sex Chromosomes in African Clawed Frogs (*Xenopus*) Suggest Some Genomic Regions Are Good at Sex Determination

## Supplemental Material for Furman, *et al*, 2016

**Files in this Data Supplement:**

- File S1 - Supporting information. (.pdf, 141 KB)
- Figure S1 - Our bioinformatics pipeline for identifying orthologous sequences used (a) Unigene and *de novo* transcriptome assemblies and (b) a modiﬁed reciprocal best BLAST hit approach to generate (c) sets of homologous sequences which were subjected to (d) quality control to ensure ungapped alignment length >300 bp, at least three ingroup species present, with at least one ingroup species with two (possibly homeologous) sequences present. We then used (e) RAxML to estimate a preliminary phy-logeny from several thousand alignments. Phylogenies were parsed for homeologs and if present (f) model selection and BEAST analysis was performed. If homeologs were still present, the longest sequence from each ortholog was retained, and (g) another model selection and BEAST analysis was performed. If two homeologs were still present the alignment was included in downstream analyses. In (a-c) colors represent different genes; paralogs have the same color. In (a-f), redundancy includes allelic variants, splice variants, non-overlapping and overlapping gene fragments, and assembly errors. For some genes, one or both homeologs were not sequenced for some individuals. (.pdf, 89 KB)
- Figure S2 - Analysis of nuclear data using B EA S T with either (a) all gene alignments concatenated together or (b) concatenated gene alignment with gapped sites removed. Individual nuclear gene trees were also analyzed with (c) MPES T as described in the methods. In (c), grey lineages have arbitrary branch lengths, CU indicates coalescent units, and numbers indicate bootstrap support. Taxa with conﬂicting phylogenetic placement within each homeologous lineage are highlighted in red; other labeling follows Fig. 1. (.pdf, 82 KB)
- Figure S3 - Bayesian analysis of mitochondrial DNA (mtDNA) alignments (after removing poorly aligned regions, see methods) with a relaxed molecular clock. This analysis produced an identical topology to that of the \*B EA S T analysis using nuclear DNA (nDNA) (Fig. 1). Labeling follows Fig. 1. (.pdf, 76 KB)
- Figure S4 - Not all SNPs are informative with respect to male versus female heterogamy. (.pdf, 50 KB)
- Figure S5 - Attempts to amplify DM-W in wild *X. clivii* were only successful in females. (.pdf, 3,442 KB)
- Table S1 - Transcriptome and GBS sequencing statistics. (.pdf, 65 KB)
- Table S2 - The primers used throughout the paper. (.pdf, 65 KB)
- Table S3 - Conditional support and median ages with the 95% bounds for various sister relationships clades summarized across the combined post-burnin posterior distributions of individual gene tree analyses (Analysis (i), methods). (.pdf, 67 KB)
