# Supplementary material for: Sequential Turnovers of Sex Chromosomes in African Clawed Frogs (Xenopus) Suggest Some Genomic Regions Are Good at Sex Determination
Source: G3 (Bethesda). 2016 Sep 7;6(11):3625–33. doi: 10.1534/g3.116.033423 (PMC5100861; doi:10.1534/g3.116.033423)
Supplement: Supplemental Material [file supp_g3.116.033423_FileS1.pdf]

Supplemental Information:

Sequential turnovers of sex chromosomes in African clawed frogs (*Xenopus*) suggest some genomic regions are good at sex determination

Benjamin L. S. Furman* **and Ben J. Evans***,[1]
*Biology Department, Life Sciences Building Room 328, McMaster University, 1280 Main Street West, Hamilton, ON L8S4K1

ABSTRACT Sexual differentiation is fundamentally important for reproduction, yet the genetic triggers of this developmental process can vary, even between closely related species. Recent studies have uncovered, for example, variation in the genetic triggers for sexual differentiation within and between species of African clawed frogs (genus *Xenopus*). Here, we extend these discoveries by demonstrating that yet another sex determination system exists in *Xenopus*, specifically in the species *X. borealis*. This system evolved recently in an ancestor of *X. borealis* that had the same sex determination system as *X. laevis*, a system which itself is newly evolved. Strikingly, the genomic region carrying the sex determination factor in *X. borealis* is homologous to that of therian mammals, including humans. Our results offer insights into how the genetic underpinnings of conserved phenotypes evolve, and suggest an important role for co-option of genetic building blocks with conserved developmental roles.

KEYWORDS
sex chromosomes
Xenopus
rapid evolution
sex chromosome turnover
SOX3

S1. SUPPLEMENTAL METHODS

S1.1. Distinguishing orthologous and homeologous sequences

Our phylogenetic analyses involved tetraploid species, and it was crucial to distinguish orthologous sequences (those that diverged from one another due to speciation events) from homeologous sequences (those that diverged from one another due to genome duplication). To accomplish this, we used a phylogenetic approach. Because genome duplication preceded speciation of the tetraploid species in our ingroup, orthologs of the different species are more closely related to one another than to homeologs of any of the species. In other words, a species will have a gene sequence that is more closely related to the gene sequence of another species, than the duplicate copy of that same gene within its own genome. Thus, we retained for analysis only those genes that had two deeply diverged lineages in at least one species (corresponding to the two sets of homeologs for that species) and assumed relationships within each of these lineages were orthologous.

To generate sequence alignments, we extracted putative coding sequence for every gene in each transcriptome assembly using Get_orfs_or_cds.py, which is part of the Galaxy Tool Shed (Cock *et al.* 2009; Blankenberg *et al.* 2014). We then used a modified reciprocal BLAST (Altschul *et al.* 1997) approach to collect homologous sequences. We BLASTed each transcriptome assembly and the *Xenopus laevis* Unigene database against the *X. tropicalis* Unigene database, and saved the top hit below a threshold *e-value* ($\leq e^{-}10$). We then blasted the *X. tropicalis* Unigene database back against each transcriptome assembly, and saved all hits below this threshold *e-value* (resulting in 15,109 *X. tropicalis* sequences with matching transcriptome sequence from the other species). We retained multiple sequences to ensure that we would capture both homeologs in the tetraploids when present, although this then required us to filter these data based on phylogenetic relationships to remove redundant sequences that were generated during transciptome assembly (see below). We retained for analysis only those BLAST matches that had at least three ingroup species with at least one species that had two sequences present (i.e. potential homeologs), leaving 7,794 sets of homologous sequences. Using a Perl script, we then generated individual sequence files for each best-BLAST result. Each file had one *X. tropicalis* ortholog and sequences from each tetraploid transcriptome matching that ortholog. We used MAFFT v.7 (Katoh and Standley 2013) to align each set of homologous sequences.

To ensure that we had enough data to make robust phylogenetic conclusions, we filtered the data sets to those that had at least 300 bp of ungapped alignment. However, some files did not meet this requirement because some sequences were short. Thus, we used a Perl script that, for alignments with less than 300 bp of ungapped alignment, would test for subsets of the data that a) match the previous taxon requirement (three ingroup + at least one species with two sequences), and b) meet the bp requirement of 300 ungapped sites. This script began by trying all combinations of taxa in an alignment that was one less than the total number of sequences (e.g. if 10 sequences in the total alignment, then all combinations of nine taxa would be tested). If none of these combinations met the taxa requirement, the file was discarded. If none of the combinations met the bp requirement (but could meet the taxa requirement), then all combinations of taxa that were two less than the total number of sequences were inspected (e.g. all combinations of eight taxa in the above example). The script would continue in this fashion, testing smaller and smaller combinations of taxa, until either both requirements were met (at which point a new alignment file would be generated with the particular combination of taxa), or the requirements could not be met and the file was discarded. The only exception to this execution were files with >15 sequences present. For these files only the total number of sequences minus one, and, if necessary, the total number minus two were tested; further combinations were deemed too computationally intensive to explore. If these files did not meet the requirements by the total number of sequences minus two, then they were discarded. If, at a given combination of taxa, multiple sets met the requirements, the script would select the combination that produced the longest ungapped alignment length. By doing this, we were able to salvage datasets that did not initially meet the bp requirement. This process left us with 3,781 gene alignments.

In addition to potential homeologs and orthologs, these alignments frequently included multiple slightly diverged but closely related sequences, which probably stemmed from allelic differences, sequence errors, misassembled transcripts, segmental duplications, and splice variants. For each alignment, we therefore used a phylogenetic approach to select representative orthologous and homeologous sequences. First, maximum likelihood trees were constructed using RAxML v.8.0.25 (Stamatakis 2014) using the GTRGAMMA model for all alignments, setting *X. tropicalis* as the outgroup, and with other parameters at the default settings. We did not perform model testing for this step because it was performed on each of two downstream phylogenetic analyses for alignments that passed an initial filter. The resulting phylogenies were then parsed using a script written in R (R Core Team 2015) and functions available in the ape (Paradis *et al.* 2004), phytools (Revell 2012), and phangorn (Schliep 2011) libraries (available with the Dryad repository doi:10.5061/dryad.00db7). This R script checked whether a tree had only two deeply diverged (putatively homeologous) sequences for at least one ingroup species. The script did this by comparing the age of the most recent common ancestors (MRCAs) of sequences within and between species; homeologs were expected to have a deeper MRCA than that of the orthologs. By identifying and comparing the MRCA of pairwise comparisons within and between species, we identified gene alignments in which there were only two lineages of sequences within the ingroup and where at least one species had one (putatively homeologous) sequence in each lineage. We then assumed that these homeologs were generated by one whole genome duplication (WGD) event prior to radiation of extant *Xenopus* species.

After building the maximum likelihood trees and filtering with the R script, we were left with 1,644 alignments with two deeply diverged *Xenopus* lineages for at least one species that we assumed were homeologous. We then built chronograms with each of these gene alignments using BEAST (Drummond *et al.* 2012). Each alignment was tested for a model of evolution using MRMODELTEST v.2 (Nylander 2004), and one MCMC chain was run for 25 million generations, using an estimated strict clock. We set the age of the root to 65 million years (my), with a standard deviation of 4.62 following (Bewick *et al.* 2012). Input files were prepared for BEAST using a Perl script. After inspecting the posterior distributions of parameter values from all analyses to ensure that stationarity had been reached for parameter estimates, we applied a conservative 25% burnin to all analyses and generated consensus trees using TREEANNOTATOR (part of the BEAST package). We then reinspected the trees with our R script, to confirm that there was still two deeply diverged lineages and identifiable homeologs, which left 1,600 alignments. At this point, because our best BLAST approach retained all matching sequences below a threshold e-value, some orthologs within each homeologous lineage were still represented by multiple sequences. We then selected the longest sequence for each species within each homeologous lineage using a Perl script. We then rebuilt the BEAST trees using two

chains run for 75 million generations (substitution models selected as before) and inspected all posterior distributions for convergence of parameter estimates (using the R package mcmcse; Flegal *et al.* 2016), and re-inspected the trees with our R script to ensure homeologs were still present.

The final data set consisted of 1,585 genes, with between five and 10 taxa (each ingroup species had two, one, or zero sequences in each alignment, depending on whether there was missing data from neither, one, or both homeologs). The individual gene alignments were 382–13,911 bp (first and third quartiles = 978–1,981 bp). The total aligned length of the data set had 2,696,030 bp, and an ungapped alignment had a total length of 788,627 bp. The proportion of missing genes for each homeolog is *X. borealis* = 0.55/0.58, *X. clivii* = 0.64/0.63, *X. allofraseri* = 0.52/0.51, *X. largeni* = 0.56/0.58, *X. laevis* = 0.14/0.14.

S2. SUPPLEMENTAL RESULTS AND DISCUSSION

S2.1. Multigene Phylogenetic Analyses of Nuclear DNA

Hereafter we refer to each set of orthologous sequences within subgenus *Xenopus* as the alpha and beta orthologs. The assignment of a particular set of orthologous sequences to each of these categories was arbitrary. The chronograms and maximum likelihood trees recovered from both concatenated data sets and the phylogeny recovered from the MPEST analysis all strongly supported a sister relationship of *X. borealis* and *X. clivii* in both the alpha and beta orthologs (Fig. S2; maximum likelihood results not shown). Similarly, the multigene *BEAST analysis also recovered strong support for this sister relationship (Fig. 1). This relationship had posterior/bootstrap support (chronograms and MPEST, respectively) of 1.0/100% in all analyses, which, in the case of the BEAST and RAXML analyses, may in part reflect overconfidence associated with analysis of concatenated data (Kubatko and Degnan 2007).

The relationships among *X. laevis*, *X. allofraseri*, and *X. largeni*, remain unresolved in all analyses. In fact, there was even conflict in the resolution of these three taxa between the alpha and beta orthologs within the same analysis (Fig. S2). For instance, in the concatenated BEAST analysis with all data, in the alpha orthologs supported a sister relationship of *X. largeni* and *X. laevis*, while the beta orthologs supported *X. largeni* and *X. allofraseri* as sister (Fig. S2). For the concatenated BEAST analysis with gapped sites removed, the alpha orthologs had the same resolution as the alpha orthologs in the full data set, but the beta orthologs supported *X. laevis* and *X. allofraseri* as sister taxa. For both concatenated chronograms, these various resolutions had posterior support 1.0 and bootstrap values >80% (and up to 100%). The MPEST analysis had the same results as the concatenated analysis with gapped sites removed, with alpha orthologs supporting *X. laevis* and *X. largeni* (93%) and beta orthologs supporting *X. laevis* and *X. allofraseri* (55%; Fig. S2). The maximum likelihood analyses also had different resolutions between the alpha and beta orthologs (results not shown). The *BEAST analysis also did not confidently resolve relationships among these three taxa, having only 0.48 posterior support for a sister relationship of *X. laevis* and *X. largeni* (Fig. 1).

S2.2. Phylogenetic discordance of individual gene trees

Using an R function, sister clade relationships were counted across the concatenated post burnin posterior distribution of each individual gene analysis (1,585 alignments; Analysis (i) in Methods). A sister relationship was counted only when at least one additional orthologous sequence was also present (to avoid inferring support when only two species were present and limit the search to topologically interesting relationships). Additionally, we collected the age of divergence for any inferred sister relationships for each tree in the posterior distribution of each gene tree analysis.

This analysis revealed considerable discordance among gene trees (Table S3). The strongest support for any clade was a monophyletic group of *X. borealis* and *X. clivii*, found in 52% and 47% of the alpha and beta orthologs, respectively, across the combined post-burnin posterior distribution of trees (with each gene having the same number of trees in the combined posterior distribution). The mean node age this group was 19.79 and 21.19 my for alpha and beta, respectively, and a large range (Table S3). A similar number of trees in the posterior distribution placed either *X. borealis* or *X. clivii* as the earliest branching $4x$=36 tetraploid *Xenopus* species. Topologies that did not support *X. borealis* and *X. clivii* as sister taxa, generally estimated an older age of divergence (Table S3), which is consistent with the hypothesis that incomplete lineage sorting contributes to the gene tree discordance. No strongly supported resolution of relationships within the clade containing *X. laevis*, *X. allofraseri*, and *X. largeni* was recovered across the gene trees, with each of the three possible sister relationships being nearly equally supported, and with similar divergence times of about 13 my (Table S3). Less than 10% of the trees in the combined posterior distribution of trees supported alternate topologies that lacked the clade containing *X. laevis*, *X. allofraseri*, and *X. largeni* and the clade containing *X. borealis* and *X. clivii*.

S2.3. The sex determining region of *X. laevis* is not homologous to that of *X. borealis*.

The Genotype By Sequencing (GBS) results indicated that the sex determining regions of *X. borealis* and *X. laevis* are non-homologous. To ensure that the region containing *DM-W* is not sex linked in *X. borealis*, we amplified a gene linked to *DM-W* (as previous attempts to amplify *DM-W* in *X. borealis* have been unsuccessful; Bewick *et al.* 2011). The gene *RAB6A* has one homolog located physically close to *DM-W* (Uno *et al.* 2013). We designed primers for both homeologs using the *X. laevis* genome v7.1 and amplified portions of both homeologs in our *X. laevis* cross. Molecular polymorphism in the homeolog of *RAB6A*, located on the unplaced scaffold 68,908, exhibited a pattern of inheritance that was consistent with sex linkage in the *X. laevis* family, with an insertion-deletion mutation in the mother inherited by all daughters and no sons (11 daughters, seven sons; Fig. S4). Conversely, parental polymorphisms in both homeologs of *RAB6A* in the *X. borealis* cross did not exhibit sex-linked inheritance; a maternal SNP on the *X. borealis* ortholog of *X. laevis* scaffold 68,908 copy was shared among daughters and sons including seven out of 11 daughters and four out of 10 sons (the father appeared to carry a null allele but, similar to *AR*, this did not affect out ability to determine a lack of sex linkage). A maternal SNP on the *X. borealis* ortholog of the other *RAB6A* homeolog in *X. laevis* (located on scaffold 19,8991) was detected in three out of six daughters and four out of eight sons, indicating that this *RAB6A* homeolog is not sex-linked. A neighbor-joining tree of these sequences confirmed the orthology and homeology of these sequences, with one homeolog in *X. borealis* grouping with the sex-linked *RAB6A* sequences of *X. laevis*, and the other *X. borealis* sequences forming their own clade.

LITERATURE CITED

Altschul, S. F., T. L. Madden, A. A. Schäffer, J. Zhang, Z. Zhang, W. Miller, and D. J. Lipman, 1997 Gapped blast and psi-blast: a new generation of protein database search programs. Nucleic Acids Res. **25**: 3389–3402.

Bewick, A. J., D. W. Anderson, and B. J. Evans, 2011 Evolution of the closely related, sex-related genes DM-W and DMRT1 in African clawed frogs (Xenopus). Evolution **65**: 698–712.

Bewick, A. J., F. J. J. Chain, J. Heled, and B. J. Evans, 2012 The Pipid Root. Sys. Biol. **61**: 913–926.

Blankenberg, D., G. Von Kuster, E. Bouvier, D. Baker, E. Afgan, N. Stoler, J. Taylor, A. Nekrutenko, *et al.*, 2014 Dissemination of scientific software with galaxy toolshed. Genome Biol. **15**: 403.

Cock, P. J., T. Antao, J. T. Chang, B. A. Chapman, C. J. Cox, A. Dalke, I. Friedberg, T. Hamelryck, F. Kauff, B. Wilczynski, *et al.*, 2009 Biopython: freely available python tools for computational molecular biology and bioinformatics. Bioinformatics **25**: 1422–1423.

Drummond, A. J., M. A. Suchard, D. Xie, and A. Rambaut, 2012 Bayesian phylogenetics with BEAUti and the BEAST 1.7. Mol. Biol. Evol. **29**: 1969–73.

Flegal, J. M., J. Hughes, and D. Vats, 2016 *mcmcse: Monte Carlo Standard Errors for MCMC*. Riverside, CA and Minneapolis, MN, R package version 1.2-1.

Katoh, K. and D. M. Standley, 2013 MAFFT multiple sequence alignment software version 7: improvements in performance and usability. Mol. Biol. Evol. **30**: 772–80.

Kubatko, L. S. and J. H. Degnan, 2007 Inconsistency of phylogenetic estimates from concatenated data under coalescence. Sys. Biol. **56**: 17–24.

Nylander, J., 2004 MrModeltest v2 distributed by author. evolutionary biology center, uppsala university.

Paradis, E., J. Claude, and K. Strimmer, 2004 {APE}: analyses of phylogenetics and evolution in R language. Bioinformatics **20**: 289–290.

R Core Team, 2015 *R: A Language and Environment for Statistical Computing*. R Foundation for Statistical Computing, Vienna, Austria.

Revell, L. J., 2012 phytools: An r package for phylogenetic comparative biology (and other things). Method Ecol. Evol. **3**: 217–223.

Schliep, K. P., 2011 phangorn: Phylogenetic analysis in r. Bioinformatics **27**: 592–593.

Stamatakis, A., 2014 RAxML version 8: a tool for phylogenetic analysis and post-analysis of large phylogenies. Bioinformatics **30**: 1312–3.

Uno, Y., C. Nishida, C. Takagi, N. Ueno, and Y. Matsuda, 2013 Homoeologous chromosomes of Xenopus laevis are highly conserved after whole-genome duplication. Heredity **111**: 430–6.
