# Supplementary material for: Sequential Turnovers of Sex Chromosomes in African Clawed Frogs (Xenopus) Suggest Some Genomic Regions Are Good at Sex Determination
Source: G3 (Bethesda). 2016 Sep 7;6(11):3625–33. doi: 10.1534/g3.116.033423 (PMC5100861; doi:10.1534/g3.116.033423)
Supplement: Supplemental Material [file supp_g3.116.033423_FigureS1.pdf]

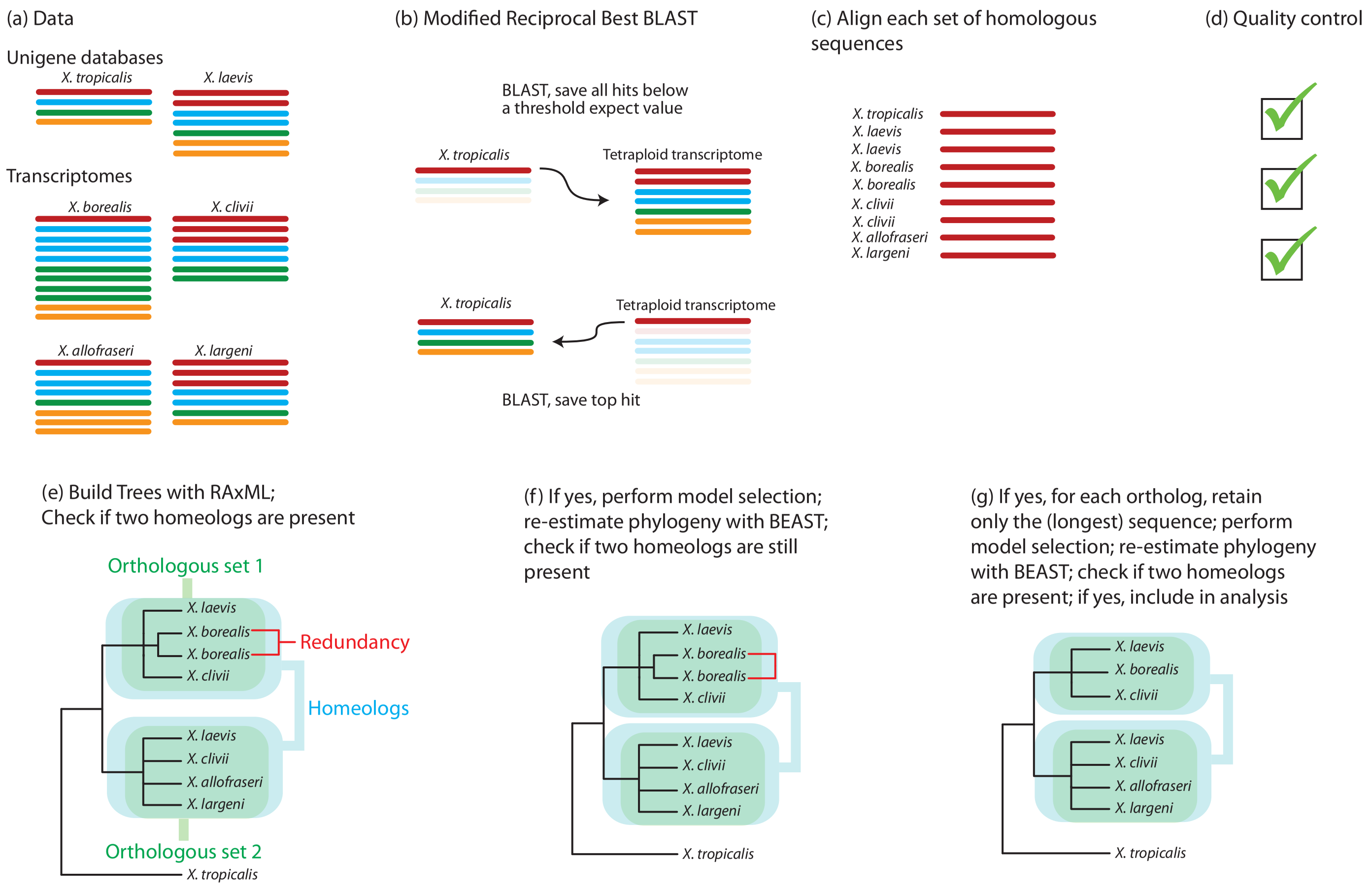

Figure S1 Our bioinformatics pipeline for identifying orthologous sequences used (a) Unigene and *de novo* transcriptome assemblies and (b) a modified reciprocal best BLAST hit approach to generate (c) sets of homologous sequences which were subjected to (d) quality control to ensure ungapped alignment length >300 bp, at least three ingroup species present, with at least one ingroup species with two (possibly homeologous) sequences present. We then used (e) RAxML to estimate a preliminary phylogeny from several thousand alignments. Phylogenies were parsed for homeologs and if present (f) model selection and BEAST analysis was performed. If homeologs were still present, the longest sequence from each ortholog was retained, and (g) another model selection and BEAST analysis was performed. If two homeologs were still present the alignment was included in downstream analyses. In (a-c) colors represent different genes; paralogs have the same color. In (a-f), redundancy includes allelic variants, splice variants, non-overlapping and overlapping gene fragments, and assembly errors. For some genes, one or both homeologs were not sequenced for some individuals.
