# Supplementary material for: Sequential Turnovers of Sex Chromosomes in African Clawed Frogs (Xenopus) Suggest Some Genomic Regions Are Good at Sex Determination
Source: G3 (Bethesda). 2016 Sep 7;6(11):3625–33. doi: 10.1534/g3.116.033423 (PMC5100861; doi:10.1534/g3.116.033423)
Supplement: Supplemental Material [file supp_g3.116.033423_FigureS2.pdf]

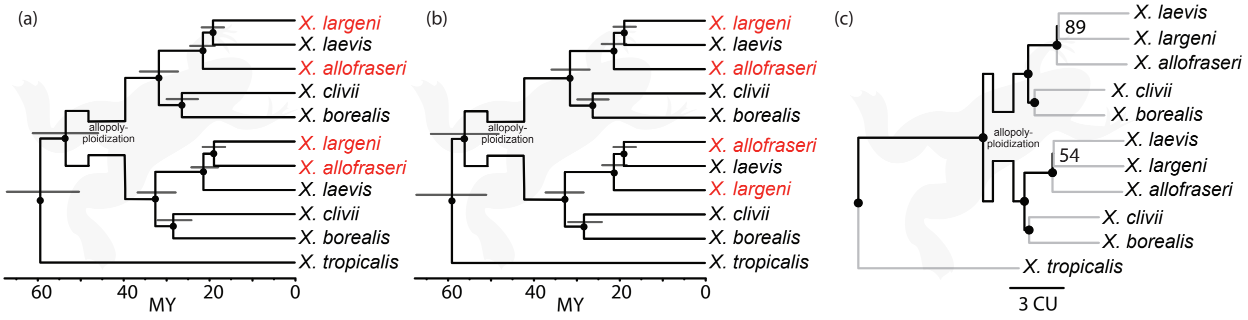

Figure S2 Analysis of nuclear data using BEAST with either (a) all gene alignments concatenated together or (b) concatenated gene alignment with gapped sites removed. Individual nuclear gene trees were also analyzed with (c) MPEST as described in the methods. In (c), grey lineages have arbitrary branch lengths, CU indicates coalescent units, and numbers indicate bootstrap support. Taxa with conflicting phylogenetic placement within each homeologous lineage are highlighted in red; other labeling follows Fig. 1
