# Supplementary material for: Sequential Turnovers of Sex Chromosomes in African Clawed Frogs (Xenopus) Suggest Some Genomic Regions Are Good at Sex Determination
Source: G3 (Bethesda). 2016 Sep 7;6(11):3625–33. doi: 10.1534/g3.116.033423 (PMC5100861; doi:10.1534/g3.116.033423)
Supplement: Supplemental Material [file supp_g3.116.033423_FIgureS3.pdf]

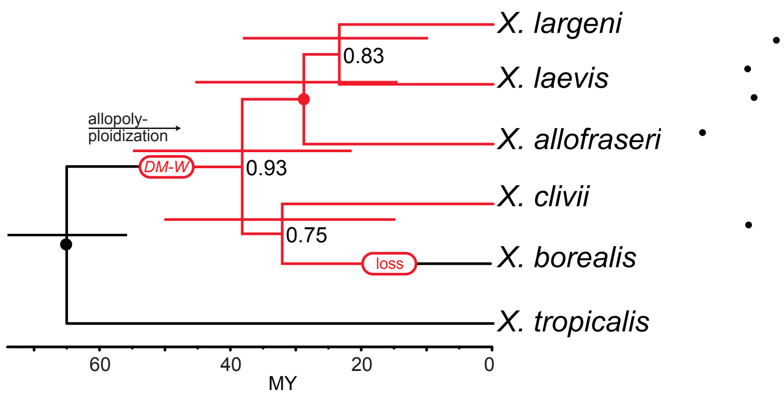

Figure S3 Bayesian analysis of mitochondrial DNA (mtDNA) alignments (after removing poorly aligned regions, see methods) with a relaxed molecular clock. This analysis produced an identical topology to that of the *BEAST analysis using nuclear DNA (nDNA) (Fig. 1). Labeling follows Fig. 1.
