# Supplementary material for: Sequential Turnovers of Sex Chromosomes in African Clawed Frogs (Xenopus) Suggest Some Genomic Regions Are Good at Sex Determination
Source: G3 (Bethesda). 2016 Sep 7;6(11):3625–33. doi: 10.1534/g3.116.033423 (PMC5100861; doi:10.1534/g3.116.033423)
Supplement: Supplemental Material [file supp_g3.116.033423_FigureS4.pdf]

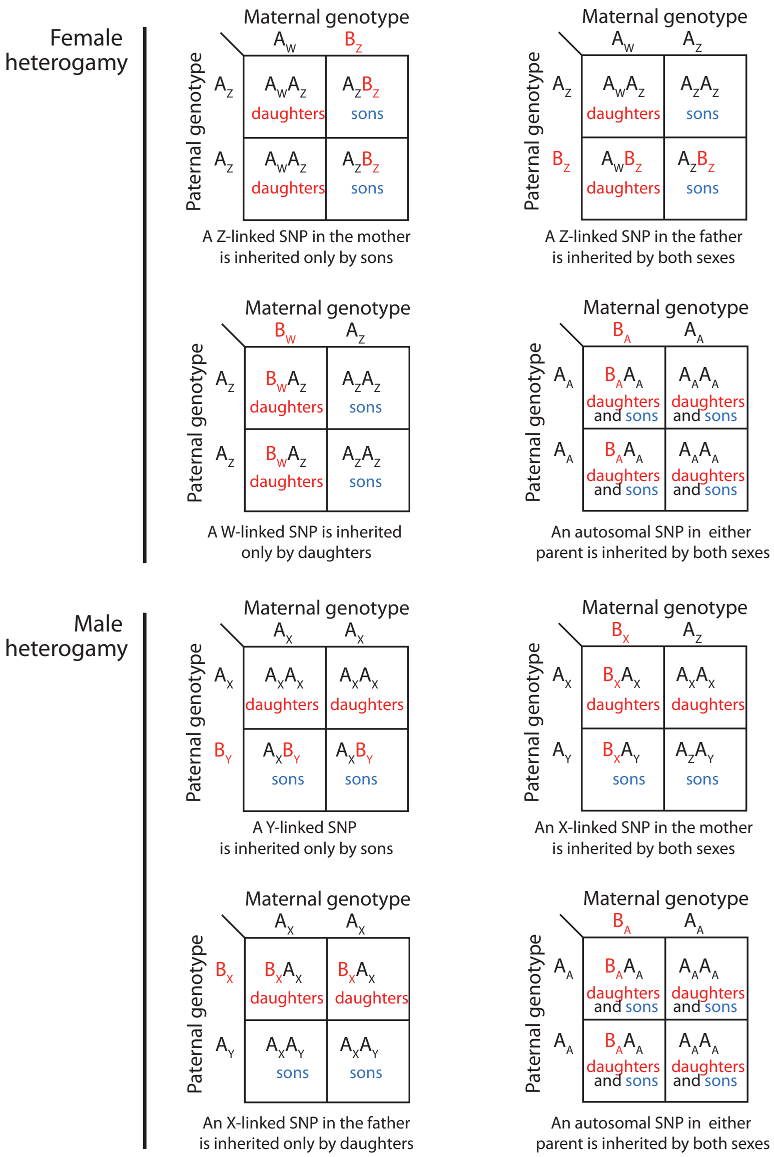

Figure S4 Not all SNPs are informative with respect to male versus female heterogamy. Diagnosis of female heterogamy requires a sex-linked SNP in the mother, and diagnosis of male heterogamy requires a sex-linked SNP in the father. For each parent, genotypes include nucleotides that are found in both parents (A) or only one (B) and that are linked to the W, Z, X, or Y chromosomes (W, Z, X, or Y subscripts respectively) or an autosome (A subscript)
