# Supplementary material for: Sequential Turnovers of Sex Chromosomes in African Clawed Frogs (Xenopus) Suggest Some Genomic Regions Are Good at Sex Determination
Source: G3 (Bethesda). 2016 Sep 7;6(11):3625–33. doi: 10.1534/g3.116.033423 (PMC5100861; doi:10.1534/g3.116.033423)
Supplement: Supplemental Material [file supp_g3.116.033423_FigureS5.pdf]

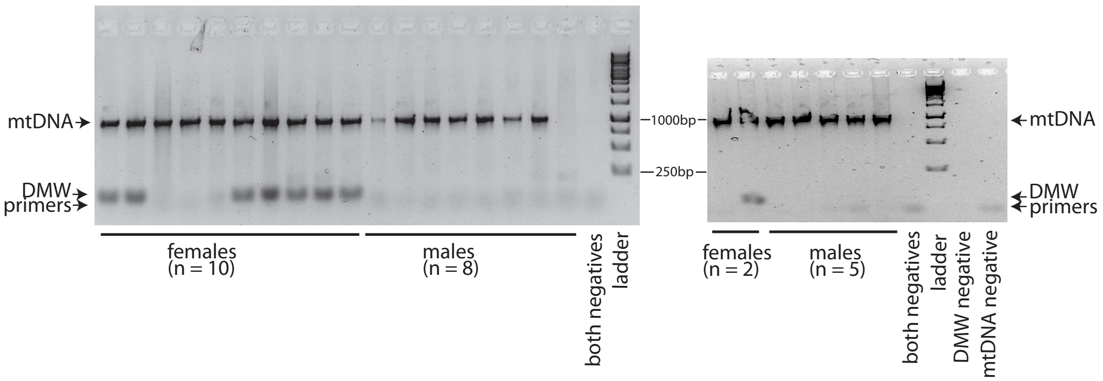

Figure S5 Attempts to amplify *DM-W* in wild *X. clivii* were only successful in females. mtDNA was used as a positive control and failed to amplify in one male.
