# Supplementary material for: Sequential Turnovers of Sex Chromosomes in African Clawed Frogs (Xenopus) Suggest Some Genomic Regions Are Good at Sex Determination
Source: G3 (Bethesda). 2016 Sep 7;6(11):3625–33. doi: 10.1534/g3.116.033423 (PMC5100861; doi:10.1534/g3.116.033423)
Supplement: Supplemental Material [file supp_g3.116.033423_TableS1.pdf]

Table S1 Transcriptome and GBS sequencing statistics. Raw sequence is number of reads; Trimmomatic is number of reads that passed our filter; Trinity is number of number of transcripts (N50 bp in brackets); Tassel values reflect reads for both lanes of sequencing (merged) and the total number of snps.

Species	Raw Sequence	Trimmomatic	Trinity	Tassel
X. allofraseri	20213893	18940455	96832 (1176)	-
X. borealis	19593759	19296751	81696 (1078)	-
X. largeni	19512126	19258973	82695 (1000)	-
X. clivii	18056373	17441776	72019 (885)	-
GBS	652154864	-	-	88996
