# Supplementary material for: Sequential Turnovers of Sex Chromosomes in African Clawed Frogs (Xenopus) Suggest Some Genomic Regions Are Good at Sex Determination
Source: G3 (Bethesda). 2016 Sep 7;6(11):3625–33. doi: 10.1534/g3.116.033423 (PMC5100861; doi:10.1534/g3.116.033423)
Supplement: Supplemental Material [file supp_g3.116.033423_TableS2.pdf]

Table S2 The primers used throughout the paper. Reverse primers provided in reverse compliment of the aligned sequence. The * denotes the additional primer combination used in the wild population samples (in text referred to as the alternate set of primers).

Gene	Primer name	Direction	Sequence
AR	begin	forward	ATGGCGGTGCACATAGGG
	down2	reverse	CGGGGGTCTCTTCGCTCT
SOX3	beta_for3	forward	GGTTTGGTCCCGGGGGCAGCGC
	beta_rev1	reverse	CTGAAGGGAAGAATGGTCGCC
	HiSeq_For5	forward	TAGGGAAGTTTTGTGCCGGGA
	HiSeq_Rev1	reverse	ACTCTGAAGGGAWGAGGGGTCG
	HiSeq_Rev5	internal reverse	GGGGTGGAAGCATTGCCCTTA
FMR1	alpha_f1_int	forward	TTTGTATTATGGTCTTTCAGGTGTATT
	alpha_r1	reverse	TTATGAATGAGCTTTTTGTTGCTGG
	alpha_f2*	forward	TGCCAACATACAACAGGCTAGGAAG
	alpha_r3*	reverse	CTAAGTTTCGTGGAACTTGTATAACATT
X. clivii DM-W	clivii_for2	forward	AATGAGGAACCATACAGCCCCAGGC
	clivii_rev2	reverse	GATTTCTGCATCGGGCACACCG
RAB6A	RAB6A_alpha_exon9_for2	forward	GCTCCTGTTAATGGCGCCCGTC
	RAB6A_alpha_exon9_rev1	reverse	CTGCTTATATATTAACAAGCCT
RAB6A	RAB6A_beta_exon9_for2	forward	GCTCCTGTTAATGTCGCCCATG
	RAB6A_beta_exon9_rev1	reverse	CTGCTTATATATTAACAAGCCC
