# Supplementary material for: Sequential Turnovers of Sex Chromosomes in African Clawed Frogs (Xenopus) Suggest Some Genomic Regions Are Good at Sex Determination
Source: G3 (Bethesda). 2016 Sep 7;6(11):3625–33. doi: 10.1534/g3.116.033423 (PMC5100861; doi:10.1534/g3.116.033423)
Supplement: Supplemental Material [file supp_g3.116.033423_TableS3.pdf]

Table S3 Conditional support and median ages with the 95% bounds for various sister relationships clades summarized across the combined post-burnin posterior distributions of individual gene tree analyses (Analysis (i), methods). The inferred sister relationship is dependent on the presence of the "condition" taxa being present (i.e. a sister relationship of *X. laevis* and *X. allofraseri* would be counted only if *X. largeni* was also in the alignment). Alpha and beta referred to the individual homeologous lineages generated by the WGD that preceded the speciation of extant *Xenopus* 4x=36 tetraploids.

homeolog	clade	condition	support	present	proportion	age	0.025	0.975
alpha	(*X. largeni*, *X. laevis*)	*X. allofraseri*	2769633	7320610	0.38	15.18	6.11	31.20
	(*X. allofraseri*, *X. laevis*)	*X. largeni*	2569313	7320610	0.35	15.10	4.46	28.47
	(*X. largeni*,*X. allofraseri*)	*X. laevis*	1806136	7320610	0.25	14.91	4.69	35.59
	(*X. clivii*, *X. borealis*)	any	3752122	7200600	0.52	22.21	7.37	40.52
	(*X. borealis*, any)	*X. clivii*	1428625	7200600	0.20	34.08	17.40	55.74
	(*X. clivii*, any)	*X. borealis*	1749065	7200600	0.25	33.64	10.54	57.36
beta	(*X. largeni*, *X. laevis*)	*X. allofraseri*	2700763	7344612	0.37	14.26	5.25	29.15
	(*X. allofraseri*, *X. laevis*)	*X. largeni*	2602804	7344612	0.35	14.90	4.87	28.99
	(*X. largeni*,*X. allofraseri*)	*X. laevis*	1899217	7344612	0.26	14.13	3.43	29.58
	(*X. clivii*, *X. borealis*)	any	3020271	6552546	0.46	23.52	6.06	41.69
	(*X. borealis*, any)	*X. clivii*	1602842	6552546	0.24	34.83	14.01	63.30
	(*X. clivii*, any)	*X. borealis*	1638245	6552546	0.25	32.65	8.85	62.56
